# Gender differences in crowd perception

**DOI:** 10.3389/fpsyg.2015.01300

**Published:** 2015-09-02

**Authors:** Yang Bai, Allison Y. Leib, Amrita M. Puri, David Whitney, Kaiping Peng

**Affiliations:** ^1^Department of Psychology, University of California, BerkeleyBerkeley, CA, USA; ^2^Department of Psychology, Illinois State UniversityNormal, IL, USA; ^3^Department of Psychology, Tsinghua UniversityBeijing, China

**Keywords:** gender differences, ensemble coding, group perception, social interaction, statistical summary

## Abstract

In this study, we investigated whether the first impression of a crowd of faces—crowd perception—is influenced by social background and cognitive processing. Specifically, we explored whether males and females, two groups that are distinct biologically and socially, differ in their ability to extract ensemble characteristics from crowds of faces that were comprised of different identities. Participants were presented with crowds of similar faces and were instructed to scroll through a morphed continuum of faces until they found a face that was representative of the average identity of each crowd. Consistent with previous research, females were more precise in single face perception. Furthermore, the results showed that females were generally more accurate in estimating the average identity of a crowd. However, the correlation between single face discrimination and crowd averaging differed between males and females. Specifically, male subjects' ensemble integration slightly compensated for their poor single face perception; their performance on the crowd perception task was not as poor as would be expected from their single face discrimination ability. Overall, the results suggest that group perception is not an isolated or uniform cognitive mechanism, but rather one that interacts with biological and social processes.

## Introduction

Humans constantly interact with groups, and many of the known perceptual and social processes reflect this important fact of human life. Our perceptions and impressions of groups of people mediate our social interactions on a daily basis—what we think and perceive about groups shapes our long-term impressions and beliefs about them and guides our moment-to-moment behavior toward them (e.g., Darwin, 1872; Park and Hastie, 1987; Jans et al., 2011). The social psychology literature on group perception has established that people's group perception process is largely affected by social influences (Linville et al., 1989; Fiske and Von Hendy, 1992), while cognitive psychologists consider group perception to be an ability which does not differ across the population of adult observers (Haberman and Whitney, 2007, 2009; de Fockert and Wolfenstein, 2009; Haberman et al., 2009). Little research, however, has investigated if both social background and cognitive abilities shape differences in people's group perception habits. Given the complexity of group perception, we propose in the current paper that individual differences (both biologically and socially constructed) influence the evaluation of a crowd. In three studies, we aim to explore whether males and females, two groups that differ cognitively and socially, also differ in the perceptual processes that underline the very first seconds of group impression formation.

Cognitive and perceptual psychologists have taken a keen interest in group perception (Haberman and Whitney, 2007; Sweeny et al., 2013). In previous literature, researchers found that when people encounter groups or crowds of people, there is too much information for the visual system to process within a short period of time (Louie et al., 2007; Whitney and Levi, 2011). The visual system overcomes this challenge by taking advantage of redundancies (Ariely, 2001; Parkes et al., 2001; Chong and Treisman, 2003, 2005; Haberman and Whitney, 2007; for reviews, see Alvarez, 2011; Whitney et al., 2014). The process of summarizing similar items or extracting summary statistical information is referred to as *ensemble coding*. Rather than analyzing each item or person individually, the visual system calculates summary statistics of similar items—for example, the average facial expression in a crowd (Haberman and Whitney, 2007, 2009; de Fockert and Wolfenstein, 2009; Haberman et al., 2009). Thus, even when individuals are unable to recall specific faces (Haberman and Whitney, 2007, 2009; Haberman et al., 2009), and unable to notice changes in crowd membership (Haberman and Whitney, 2011), they are still able to calculate the summary statistics of the crowd (i.e., mean emotion or identity) using ensemble coding mechanisms. The common assumption shared by previous cognitive researchers is that group perception is a cognitive ability that is similar across all adult perceivers and is influenced by the object features of the crowd, e.g., the size of the crowd (Haberman and Whitney, 2009), the duration of the displaying faces (Haberman et al., 2009), the orientation of the crowd (Haberman and Whitney, 2007, 2009; Haberman et al., 2009), etc.

Groups are clearly at the heart of many social psychological phenomena (Hamilton and Gifford, 1976). Therefore, research on group impressions is ubiquitous: for example, group entitativity (e.g., Campbell, 1958; Lickel et al., 2000), homogeneity (e.g., Rothbart and Park, 2004; Hamilton, 2007), stereotyping (Schaller and O'Brien, 1992; Hamilton and Sherman, 1994), as well as important self-relevant processes such as self-identity (Turner, 1985; Spears et al., 2001), out-group threat (Fiske, 2002; Yuki and Yokota, 2009), conformity signals (Asch, 1956; Cialdini and Trost, 1998), or even urgent social information (Helbing et al., 2000) are all based on the outcomes of group perception. Differing from cognitive psychologists, social psychologists assume that group perception is a high-level process which is diverse across individuals–emphasizing social influences on people's evaluation, inference, and attribution processes (Linville et al., 1989). For instance, a substantial amount of previous research has found that people tend to perceive the group they belong to (their in-group) with more variance (e.g., Park and Rothbart, 1982; Linville et al., 1989; Ostrom and Sedikides, 1992). In addition, one's power status (Guinote et al., 2002), cultural orientation (Spencer-Rodgers et al., 2007), and ethnic background (Chen and Hamilton, 2012; Chen et al., 2014) were also found to influence one's group perception habits.

Although these methods and analyses in both perceptual and social psychology are valuable, their assumptions are not complete. On one hand, group perception is not solely a high level process–low level visual discrimination of facial identity, ethnicity, and emotion shapes the impressions we form of other people, and social communication depends on these impressions as well (Asch, 1946; Ekman, 1973; Todorov, 2012). On the other hand, crowd perception necessarily fluctuates among groups of individuals due to their different social and cognitive backgrounds (Park and Rothbart, 1982; Guinote et al., 2002; Spencer-Rodgers et al., 2007; Leib et al., 2012). As a first step toward testing how both social and cognitive factors influence our group perception process, we chose gender as a test bed since it has long been regarded as a group factor which predicts various individual differences within the fields of both social psychology and cognitive psychology (Fiske, 2010). The origin and scope of psychological gender differences are some of the most challenging and fascinating questions for both social and cognitive psychologists, and require interdisciplinary investigations that go beyond simple contrasts between the genders (Eagly and Wood, 1999; Wood and Eagly, 2002). For cognitive psychologists, the innate biological differences such as steroidal gonadal hormone difference (e.g., Hines, 1982; Collaer and Hines, 1995), genetic disparity (e.g., Heath et al., 1999), and cognitive ability gap (e.g., Hyde and Linn, 1988; Miller and Halpern, 2014) reportedly contribute to the differences found between females and males. Social psychologists, on the other hand, tend to focus on social construction explanations, where the importance of societal role differentiations for females and males (e.g., Bohan, 1993) provides an explanation for observed discrepancies between the two genders.

Previous research has indicated that, in general, females perform better than males on single face perception tasks—including tasks using affective and non-affective faces (e.g., Rehnman and Herlitz, 2006; McBain et al., 2009; Heisz et al., 2013). This difference can be explained from both the cognitive and social perspectives. Cognitively, females' superior performance on single face perception tasks could be attributed to their innate perceptual advantages, such as low spatial frequency detection or better holistic processing (McBain et al., 2009). Socially, historical gender differences, such as males participating in larger social networks and females participating in smaller social networks, may also influence the way males and females view groups vs. individual faces (for review, see Belle, 1989; Benenson, 1990). However, no study to date has tested gender differences in group identity perception. On the one hand, it is reasonable to hypothesize that superior single face perception will contribute to performance on crowd averaging, as the crowd average is necessarily comprised of individual face assessments. On the other hand, however, from a social or cognitive perspective, group perception is also not simply a sum of single face judgments (Alvarez, 2011; Whitney et al., 2014). Previous cognitive research has found that ensemble percepts may slightly compensate for single item perception (Leib et al., 2012). In the present experiment, we investigated whether males and females differ in ensemble face perception and whether or not this difference interacts with their abilities to discriminate single faces. We hypothesize that consistent with previous findings, females will outperform males on single face perception which will largely contribute to their crowd perception performance. We also hypothesize that, compared to females, males will exhibit their own advantage in group perception. This is consistent with previous findings in the cognitive literature that ensemble perception performance among prosopagnosics is far better than would be expected from their single face perception ability (Leib et al., 2012), and social observations that males' social network size is larger than females' on average (Benenson, 1990). We hypothesize that males' ensemble coding performance will be better than is predicted by their single face discrimination and even exceed females' group integration performance. In Experiment 1, we tested gender differences in homogeneous and heterogeneous crowd perception using an identity averaging task (Haberman and Whitney, 2007, 2009; Haberman et al., 2009). In Experiments 2a and 2b, we tested gender differences in single face perception (Chong et al., 2008; Haberman et al., 2009) and in ensemble coding for both redundant (crowds with repeated face sets and same variance) and non-redundant (crowds with non-repeated faces and changing variance) crowds.

This project was approved by both the University of California Berkeley Committee for the Protection of Human Subjects and Tsinghua University Committee for the Protection of Human Subjects. Written consent from all individuals for their voluntary participation was obtained before beginning of the study.

## Experiment 1

### Participants

Twenty-three undergraduate students (12 male; *M* = 20.17 years, *SD* = 1.15 years) at Tsinghua University in China participated in the experiment in exchange for optional course credit. Participants gave written informed consent according to the guidelines of the local research ethics committee.

### Materials

**Figure 1:**
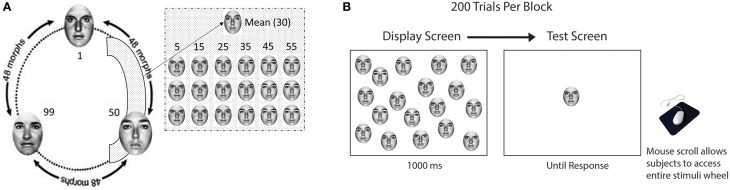
**Materials and procedure for Experiment 1. (A)** One hundred and forty-four faces morphed between three identities to create one wheel of 147 faces (female upright wheel presented here). In each trial, the computer randomly chose a mean face (face 30 in the figure) and 18 faces either repeating the mean 18 times (homogeneous condition) or ±5, ±15, and ±25 steps away from the mean (heterogeneous condition). **(B)** In the study, participants saw a crowd for 1000 ms before they were presented with a test face on the screen. Participants were asked to use the mouse to scroll through the wheel of faces and “adjust” the test face. When the adjusted test face matched with their average estimation of the previously seen crowd, they would left click their mouse and move to the next trial.

In Experiment 1, our stimuli consisted of two arrays of faces. Each array contained 147 faces, generated by morphing together photographs of three different individuals (A, B, and C), each with a neutral expression (Figure 1). One array was morphed from three grayscale female photos from the Ekman gallery (Ekman and Friesen, 1976), while the other array was morphed from three grayscale male photos from the same gallery. We used the program Morph 2.5 (Gryphon Software, San Diego, CA) to linearly interpolate each of the three pairs of original faces (A and B, B and C, C and A) to create 48 morphs per pair, yielding a “circle” of 147 neutral faces with no end or beginning (144 morphed pictures + 3 original = 147 faces total). We repeated this procedure to create an additional array of male faces (see Figure 1). During the experiment, participants viewed 18 faces drawn from either the male or female array. The faces were shown in a grid pattern on the computer screen. In two blocks, we displayed faces upright, while in two other blocks we displayed faces inverted counterbalanced across subjects. Subjects performed the experiment in a darkened room, seated at a distance of 80 cm from the monitor. For a more detailed description of stimulus creation and viewing conditions, please see the Supplementary Material.

### Procedure

For each trial, participants were presented with a crowd of 18 faces for 1000 ms. The 18 faces were drawn from the stimulus set and centered around a randomly chosen face (see Figure 1). After the crowd of faces disappeared, a single test face, which was randomly chosen from the entire wheel, was presented centrally on the screen (see Figure 1). Participants were instructed to “adjust” the test face until it looked exactly like the average identity of the previous 18-face crowd display. By moving the mouse left or right, participants viewed a face that seamlessly transformed into different identities (see Supplementary Video 1). Participants could click (using the left mouse button) on the face that matched their precise estimation of the mean. This allows us to objectively measure participants' ability to mentally represent the average identity of the crowd. Participants adept at mentally representing the average identity would select a morphed face that was quantitatively close to the mean, whereas participants with a diminished capacity to represent the average identity would select a face that was quantitatively further from the mean of the crowd. The next trial began 200 ms after the button was pressed. Sets with consistent face gender (female or male) and orientation (upright or inverted) were presented in randomized blocks of 200 trials, in different orders for each participant.

Half of the displays in each block were heterogeneous crowds comprised of six identities, each repeated three times, for a total of 18 faces in the crowd. The six identities in each crowd were ±5, ±15, and ±25 steps away from the mean face in morph units. The mean face was randomly chosen from the 147 face array, but was not included in the display. The other half of the trials in a block contained homogeneous displays in which all 18 faces were the same. This homogeneous condition tested participants' recognition of a single identity (all the faces displayed were identical). In other words, the homogeneous condition is similar to the heterogeneous condition—except it does not require participants to average faces in order to respond correctly.

### Analysis

We analyzed participants' accuracy for each trial, using the following equation: Error = Actual Mean of Display (in morph units)-Participant's Response (in morph units). The units used in all equations are morph units. Importantly, we took the mean of the absolute error in each condition and obtained the Average Estimation Error (AEE).

### Results and discussion

**Figure 2:**
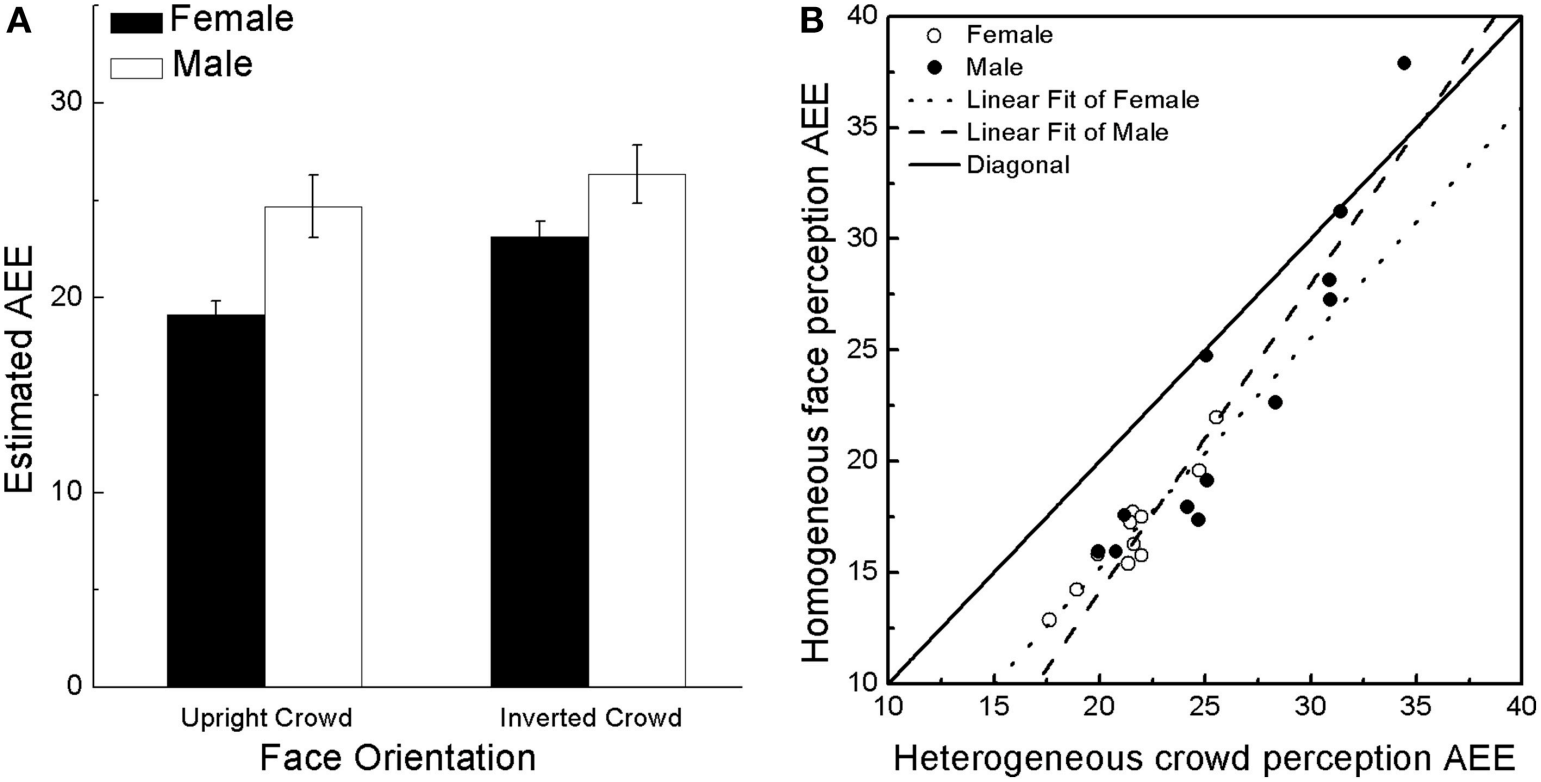
**Results of Experiment 1. (A)** Males' and females' estimated AEE for upright crowds and inverted crowds. Error bars represent bootstrapped ± 1 SD. **(B)** Relationship between single identity discrimination (homogeneous upright face condition) and crowd face perception (heterogeneous upright face condition) for males and females in Experiment 1.

We conducted a 2 (subject gender: female vs. male) × 2 (picture orientation: upright vs. inverted) × 2 (display condition: heterogeneous vs. homogeneous) × 2 (picture gender: female vs. male) ANOVA. Subject gender was a between-subject variable. Consistent with previous research (Haberman and Whitney, 2009; Leib et al., 2012), the ANOVA revealed a significant main effect of picture orientation: participants were significantly better in estimating the mean identity for upright faces compared to inverted faces [*F*_(1, 21)_ = 23.56, *p* < 0.001, $\eta_{p}^{2}=0.53$]. The variance also had a significant effect on subjects' results; subjects performed much better when the displays were homogeneous [*F*_(1, 21)_ = 82.37, *p* < 0.001, $\eta_{p}^{2}=0.80$]. Furthermore, as we predicted, we found a significant effect of subject gender [*F*_(1, 21)_ = 6.27, *p* < 0.05, $\eta_{p}^{2}=0.23$]; females' AEE for mean identity (*M* = 21.14, *SE* = 1.26) was smaller (more precise) than male subjects' AEE (*M* = 25.52, *SE* = 1.20). The display variance × participant's gender interaction was not significant [*F*_(1, 21)_ = 1.21, *p* = 0.28 > 0.05], suggesting that females were overall more accurate in perceiving single face and ensemble coding group of faces. The orientation × participants' gender interaction was marginally significant [Figure 2A; *F*_(1, 21)_ = 4.07, *p* = 0.057, $\eta_{p}^{2}=0.16$]. Simple effect analysis of this interaction revealed that when the display faces were upright, females performed significantly better than males [*F*_(1, 21)_ = 8.82, *p* < 0.01, $\eta_{p}^{2}=0.30$]; when the display faces were inverted, the two genders' performance was only marginally different [*F*_(1, 21)_ = 3.12, *p* = 0.092, $\eta_{p}^{2}=0.13$]. This result suggests that females are not simply better at identifying simple shape or color contrasts in the crowd of faces (the shapes and color contrasts are still available when the crowd is inverted). Instead, females are actually better at ensemble coding a crowd of faces as they would be presented in typical social settings (upright). In addition, the interaction between participants' gender and stimulus gender was not significant [*F*_(1, 21)_ = 0.40, *p* = 0.532, $\eta_{p}^{2}=0.02$], excluding the possible own-gender effect which might lead to the observed gender difference.

The ANOVA result revealed that females performed better than males in the homogeneous upright crowd condition and in the ensemble group perception condition. However, the exact nature of females' perceptual advantage remained unclear. To further examine the relationship between single face perception ability and ensemble coding ability for both genders, we examined the correlation between the heterogeneous and homogeneous conditions (Figure 2B). We fitted all subjects' AEE with a linear function Y = *b*X + *c*, in which each subject's AEE for the homogeneous upright condition was Y and AEE for the heterogeneous upright condition was X. The slope parameter (*b*) of the fitted line reveals the relationship between sensitivity to the heterogeneous upright face condition (mixture of identities) and sensitivity to the homogeneous upright face condition (one identity). A steeper slope indicates that individuals tend to perform better in the ensemble coding condition relative to their homogeneous identity discrimination. The result showed that males had a steeper slope compared to females (*b*_male_ = 1.38; *b*_female_ = 1.04). Although a permutation test^1^ showed that the difference between the slopes for the two genders was not significantly different (*p* = 0.149), the males' slope coefficient was greater than that of the females, suggesting that despite their relatively poor sensitivity to single identities, male subjects' sensitivity to crowd identity might be slightly increased.

Consistent with previous research showing that females were better at discriminating single faces (e.g., Rehnman and Herlitz, 2006; McBain et al., 2009), these data suggest that females are relatively better at discriminating a single identity (homogeneous condition in this case). Furthermore, these results are the first to show a novel gender difference in group perception. Our most robust finding is that females perceive the mean identity of a crowd (heterogeneous condition) more accurately than males.

## Experiment 2a

In Experiment 1, it was unclear whether females' relatively better performance was due to their precision in single face discrimination or due to an advantage in integrating the faces in the display (i.e., ensemble coding). One limitation of Experiment 1 was that it did not contain a true single face discrimination task. In the baseline homogeneous condition of Experiment 1, participants viewed a crowd of faces (albeit the same face repeating 18 times). Another limitation is the variability of the crowds displayed in Experiment 1. Previous research has found that high variation in the heterogeneous display negatively impacts ensemble coding performance (Haberman and Whitney, 2009). It is therefore possible that the variance between identities in the heterogeneous crowd of faces in Experiment 1 was higher than ideal for ensemble coding experiments. Additionally, the sample size (23) may be too small to draw definitive conclusions about the differences in performance between males and females. Therefore, by reducing the variance of the displayed faces and creating a true “single face” condition, Experiment 2a aimed to replicate the ensemble coding result of Experiment 1, and to determine whether females' advantage was due to superior single face perception or superior integration abilities.

### Participants

Sixty undergraduate students at Tsinghua University in China participated in exchange for an optional course credit. Two subjects did not complete all conditions; their data was excluded from the analysis. The remaining 58 were comprised of 28 females and 30 males (*M* = 18.81 years, *SD* = 1.01).

### Materials

The stimuli were identical to the stimuli used in Experiment 1, except that we varied the number of faces displayed. For each trial, participants were presented with 1, 4, 8, or 12 faces. Trials with varying set sizes were randomly interleaved. Each face displayed was 3 cm by 3.7 cm, subtending 2.86° of the visual angle. All stimuli were viewed on a 19 inch monitor with resolution of 1280 × 1024 pixels, with a 75 Hz refresh rate. Subjects performed the experiment in a darkened room, seated at a distance of 80 cm from the monitor.

### Procedure

For Experiment 2a, face gender (female or male) and orientation (upright or inverted) was presented in blocks of 200 trials each. In each trial, the display included 1, 4, 8, or 12 faces for 500 ms. For the real single face condition, a random face was chosen and displayed on the screen for each trial. For the crowd condition of 12 faces, just as before, the mean face was randomly chosen from the array of 147 faces and never shown to participants; the faces displayed were three and nine steps away from the mean face in either direction, with a consistent variance. This design yields a crowd of 12 faces with four distinct identities, each repeated three times. Crowds consisting of four or eight faces contained repeated identities one or two times, respectively. As in the first experiment, participants used the method of adjustment to report the mean identity of the crowd. The next trial began 200 ms after the participant registered their response.

### Results and discussion

Participants' AEE under different conditions was measured as described in Experiment 1. We conducted a 2 (subject gender: female vs. male) × 2 (picture orientation: upright vs. inverted) × 4 (display condition: 1, 4, 8, or 12 displayed faces) × 2 (picture gender: female vs. male) ANOVA. Only subject gender was a between-subject variable. The results were similar to Experiment 1. The ANOVA revealed a significant main effect of picture orientation; participants were significantly better in estimating the mean identity for upright faces compared to inverted faces [*F*_(1, 56)_ = 90.30, *p* < 0.001, $\eta_{p}^{2}=0.62$]. The number of display face(s) also significantly influenced people's sensitivity to crowd identity [*F*_(3, 56)_ = 8.57, *p* < 0.001, $\eta_{p}^{2}=0.13$]. Bonferroni *post-hoc* tests suggested that participants perceived the single face more accurately than they ensemble coded a group of faces, regardless of group size (*M*_1face_ = 23.44, *SE* = 0.48; *M*_4faces_ = 24.79, *SE* = 0.50; *M*_8faces_ = 24.52, *SE* = 0.56; *M*_12faces_ = 24.63, *SE* = 0.58). Consistent with previous research (Haberman et al., 2009), the interaction between display face number and face orientation was significant [*F*_(3, 168)_ = 4.24, *p* < 0.01, $\eta_{p}^{2}=0.07$]. The number of faces in the display had a significant impact on participants' estimates in the upright face condition [*F*_(3, 171)_ = 14.18, *p* < 0.001, $\eta_{p}^{2}=0.20$], but had no effect when the faces were inverted [*F*_(3, 171)_ = 1.73, *p* = 0.163, $\eta_{p}^{2}=0.03$].

Consistent with the results of Experiment 1, we found a significant main effect of subject gender [*F*_(1, 56)_ = 4.53, *p* < 0.05, $\eta_{p}^{2}=0.08$]. Female participants (*M* = 23.29, *SE* = 0.72) were more accurate at estimating the mean face compared to male subjects (*M* = 25.41, *SE* = 0.69), and there was no significant interaction between subject gender and the number of display faces [*F*_(3, 168)_ = 0.98, *p* = 0.40 > 0.05]. Although the interaction between face orientation and participants' gender did not reach significance [*F*_(1, 56)_ = 2.66, *p* = 0.109, $\eta_{p}^{2}=0.05$], a simple effect analysis revealed that females outperform males only when the display faces were upright [*F*_(1, 56)_ = 6.03, *p* < 0.05, $\eta_{p}^{2}=0.10$], while males' and females' performance did not differ when the display faces were inverted [*F*_(1, 56)_ = 2.10, *p* = 0.152, $\eta_{p}^{2}=0.04$]. Again, participants' gender did not significantly interact with the stimulus gender [*F*_(1, 56)_ = 0.12, *p* = 0.732, $\eta_{p}^{2}=0.002$].

**Figure 3:**
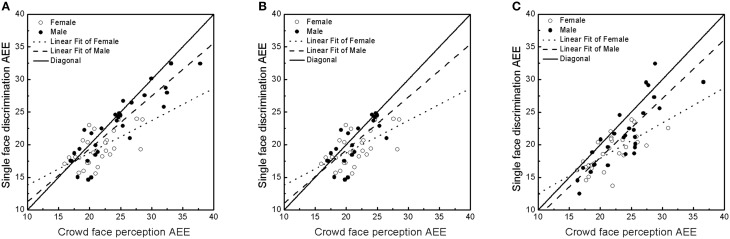
**Results of Experiments 2a and 2b. (A)** Relationship between single face discrimination and perception of crowds of redundant upright faces (crowds contained repeated pictures of four identities on each trial) for males and females in Experiment 2a. Crowd face perception AEE was the average AEE for the 4, 8, and 12 face conditions. **(B)** Relationship between single face discrimination and redundant upright crowd face perception for all females and the subset of males who had an equivalent single face perception as females in Experiment 2a. **(C)** Relationship between single face discrimination and non-redundant upright crowd face perception for males and females in Experiment 2b.

In this experiment, in contrast to the single face condition, the other three conditions required participants to ensemble code the crowd in order to provide a more precise average estimation. In order to test the relationship between single face detection and ensemble coding of crowds, we collapsed the 4, 8, and 12 upright face conditions together and performed the same linear regression described in Experiment 1 (Figure 3A) using each subject's AEE for the single upright face condition as Y and AEE for the multi upright face condition (collapsed across 4, 8, and 12 upright faces conditions) as X. The results again showed that males had a steeper slope compared to females (*b*_male_ = 0.811; *b*_female_ = 0.495), and a permutation test showed that this difference was significant (*p* < 0.001). This indicates that, despite their relatively poor sensitivity to single faces, male subjects' sensitivity to crowd identity was relatively high.

In a complementary analysis, we examined the proportion of subjects that had a smaller AEE in the crowd perception condition compared to their performance in the single face discrimination condition. More precision in heterogeneous crowd identity perception than single face identity perception indicates that more than one face has been integrated into analysis when perceiving the crowd (the only way to perceive heterogeneous crowds more precisely than a single face is by averaging, which cancels out noise). We found that 37% of male subjects had a smaller estimation error in the crowd perception condition compared to their performance in the single face discrimination condition, whereas only 25% of females had a smaller estimation error in the crowd perception condition compared to their performance in the single face discrimination condition (male vs. female difference: *p* = 0.096, permutation test). A relatively smaller (better) estimation error in the crowd vs. single face condition suggests that subject performance in the single face condition is not the absolute limit of subject performance in the crowd condition. That is, some subjects perceived crowds *more precisely* than they perceived single faces, and this held more widely across the male participants than across the female participants. Figure 3A depicts the larger proportion of males that performed adequately during ensemble coding tasks, despite relatively poor single face perception performance.

In order to test whether the observed gender differences (the slope difference in Figure 3A) was caused by a difference in the two genders' abilities in the single face condition, we re-conducted the analysis using only data from males who showed similar single face perception performance to females. That is, we used only male subjects whose AEE was in the range of the females' AEE for the upright single face condition, ensuring equivalent single face estimation performance between female and selected male participants. The analysis confirmed that the slope of male subjects was steeper than that of female subjects (Figure 3B; *b*_male_ = 0.813; *b*_female_ = 0.495). A permutation test showed that those males who performed as well as females in the single face perception condition still tended to have a larger slope than female subjects (*p* = 0.06), suggesting that the observed gender difference was not driven by a subset of males who performed poorly.

Experiment 2a provides several important insights. First of all, we replicated our main finding in Experiment 1 that females perform better than males in both the single face discrimination task and the ensemble coding task. Additionally, the proportion test and slope test above both provide evidence showing that although males' *absolute* sensitivity is relatively lower than females' sensitivity (males' overall estimation error was larger than females'), males still achieve adequate ensemble coding performance. Essentially, male subjects' performance in the crowd condition was not as poor as would be expected from their single face discrimination. Even when controlling for the male subjects' abilities in the single face discrimination condition, we still observed a steeper slope in male subjects' performance. These results suggest that while females robustly perceive crowds with higher precision, males might have their own perceptual advantage in the ensemble coding process.

## Experiment 2b

In Experiment 2a, the different set sizes shared the same variance. Thus, the information conveyed by the 4, 8, and 12 set size displays was identical—except that the 8 and 12 set size displays contained redundant faces. Previous research indicated that redundant faces within crowds improved participants' ensemble coding performance (Haberman and Whitney, 2010). In order to minimize this redundancy effect, we repeated the experiment using crowds of faces with randomly chosen variance (see Supplementary Figure 1). Thus, Experiment 2b aimed to replicate the results of Experiment 2a, while allowing participants to view faces with a fluctuating variance. In other words, in some trials participants viewed crowds with a variance of 0 while in other trials participants viewed crowds with a variance of 12.73, and many values of variance in between. The variance changed according to the randomly chosen faces^2^ (see Supplementary Figure 1).

### Participants

Fifty eight undergraduate students at Tsinghua University in China participated in exchange for an optional course credit. Among all the subjects, two subjects did not complete all conditions and their results were excluded. The remaining 56 were comprised of 28 females and 28 males (*M* = 20.59 years, *SD* = 1.47).

### Materials and procedure

The stimuli and settings were identical to the stimuli used in Experiment 2a, with one significant difference. In Experiment 2b, the variance of the display was not restricted. For each trial, the program first chose 12 faces. The 12 faces were comprised of four faces that were either three or nine units away from the randomly selected mean face in both directions, repeated three times (similar to the display depicted in Figure 1A). Next, we randomly presented 1, 2, 4, or 8 faces out of the chosen 12-face set. Thus, the variance in the displayed faces was not consistent throughout the experiment as it was in Experiment 2a. In Experiment 2b, the displayed face variance fluctuated according to the faces selected for each trial. This minimized the effects of redundancy. All other procedures were the same as those in Experiment 2a.

### Results and discussion

Similar to Experiment 2a, we conducted a 2 (subject gender: female vs. male) × 2 (picture orientation: upright vs. inverted) × 4 (display condition: 1, 2, 4, or 8 visible faces) × 2 (picture gender: female vs. male) ANOVA. Only subject gender was a between-subject variable. Similar to previous research and the results of Experiment 1, the ANOVA revealed a significant main effect of picture orientation. Participants were significantly better in estimating the mean face for upright faces compared to inverted faces [*F*_(1, 54)_ = 81.86, *p* < 0.001, $\eta_{p}^{2}=0.60$]. The number of display face(s) also significantly influenced ensemble coding performance [*F*_(3, 162)_ = 25.23, *p* < 0.001, $\eta_{p}^{2}=0.32$]. Consistent with the results of Experiment 2a, Bonferroni *post-hoc* tests revealed that participants were more accurate in single face discrimination compared to ensemble coding a group of faces, regardless of the group size (*M*_1face_ = 22.04, *SE* = 0.53; *M*_4faces_ = 24.09, *SE* = 0.51; *M*_8faces_ = 24.13, *SE* = 0.53; *M*_12faces_ = 24.45, *SE* = 0.52). In addition, we again found a significant effect of subject gender [*F*_(1, 54)_ = 5.32, *p* < 0.05, $\eta_{p}^{2}=0.075$], which is consistent with our previous results. Female participants (*M*_female_ = 22.55) were more accurate at estimating the mean face compared to male subjects (*M*_male_ = 24.80), and there was no significant interaction between subject gender and display face number [*F*_(3, 162)_ = 0.37, *p* = 0.77 > 0.05]. Consistent with results of Experiment 1 and 2a, participants' gender did not interact with the stimulus gender [*F*_(1, 54)_ = 2.68, *p* = 0.107, $\eta_{p}^{2}=0.05$].

We collapsed the AEE across the 2, 4, and 8 upright face conditions and performed the same slope fitting test again, as in Experiments 1 and 2a. The results again showed that, compared to females, males had a steeper slope (Figure 3C; *b*_male_ = 0.903; *b*_female_ = 0.541). A permutation test showed that the males' slope was significantly steeper than females' slope (*p* < 0.05). The slope of the subgroup of males who had equivalent single face perception performance to females (*b*_male_ = 0.675) was compared to the slope of the full set of females (*b*_female_ = 0.541). Although a permutation test did not find a significant difference between the two slopes (p = 0.11), the pattern suggests a trend that males who performed as well as females in the single face perception condition tended to have steeper slopes. Again, a higher proportion of males tended to perform better in the crowd than in the single face condition (Male = 21.4%; Female = 10.7%, *p* = 0.063).

In sum, Experiment 2b replicated the result of Experiment 2a, even though the faces in each set were not necessarily redundant.

## Simulation results

**Figure 4:**
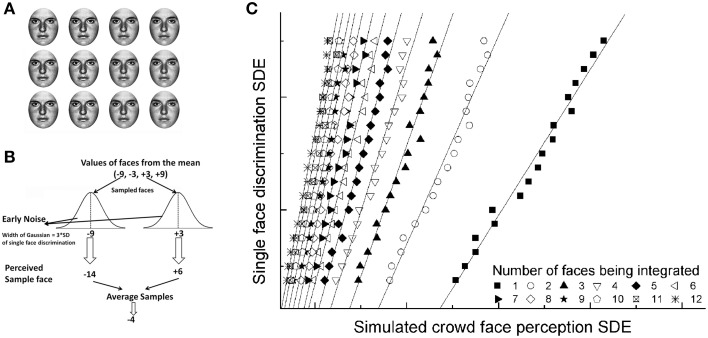
**Simulation result. (A)** Twelve faces were chosen by the program to replicate the empirical experiment (the faces were −3, −9 and +3, +9 units away from the randomly selected mean). **(B)** A simulated integration process in which participants averaged 1–12 (two in the figure) noisily encoded faces to estimate the mean. Noise was determined by the standard deviations of participants' error distributions for the single face perception condition. **(C)** Linear fit of subjects' simulated crowd face perception sensitivity as a function of single face discrimination. We depict increasing integration conditions ranging from the smallest set (one face) to the largest set (12 faces) from right to left. We observe a pattern of steeper slopes with increased integration, consistent with conclusion that males integrated more faces than females, despite their relatively poor overall ensemble coding performance.

Many ensemble coding experiments simulate the number of faces/objects integrated into the ensemble percept (Haberman and Whitney, 2010; Leib et al., 2012; Im and Halberda, 2013). This follow-up analysis allows researchers to investigate specifics of the ensemble coding mechanism when large numbers of items are integrated into the ensemble percept. This is important because while statistical averaging can technically include as little as two faces from the crowd, in natural settings integrating more faces would yield the greatest information about the crowd. Thus, we investigated whether previously observed patterns between males and females remain robust under such simulations. Specifically, how does single face sensitivity correlate with crowd sensitivity when larger numbers of faces are integrated from the display? The simulation contained the following four steps: (1) We simulated the value (relative to the mean) of the faces displayed—in this case (−9 to +9) (see Figure 4A). (2) Next, we simulated the number of faces (*N*) sampled from the display. Each face was drawn from a Gaussian distribution centered at the display face value. (3) Next, noise was added to the chosen face values based on the range of the participants' empirical data in the single face condition (Figure 4B shows a description of this step). (4) *N* face values were averaged in a noise-free integration step. (5) Error was determined by subtracting this average from the true mean of the crowd^3^. We simulated 1000 bootstrapped trials and the standard deviation of the error distribution was calculated and regarded as the simulated group perception performance. For each simulation, we iteratively chose a higher *N* to simulate performance when participants systematically integrated more and more faces in the display.

We compared the participants' empirical single face discrimination score to the simulated ensemble coding performance using a linear regression test (Figure 4C). The analysis indicates that as more faces were integrated into the mean judgment, the slope became steeper. This pattern suggests that it is not only the discrimination of single faces that contributes to performance, but also the integration process that allows participants to successfully evaluate the mean identity of the crowd. Importantly, we observed a significant difference in males' and females' slope values. Males exhibited a steeper slope compared to females as the number of faces integrated increased. The gender slope difference combined with the simulation result indicate that although males' performance was lower overall, they may be integrating a larger number of faces into their ensemble percepts.

The simulation is an exploratory analysis conducted *post-hoc*. Nonetheless, it offers some potential insight into the ensemble coding process of both males and females. Females are superior at ensemble coding, but males are theoretically still able to integrate a large number of faces into their ensemble percept, despite their poor single face performance. This may even suggest that ensemble coding serves as a compensatory mechanism for males.

## General discussion

The current study is the first to examine how males and females might differ in their initial perceptions of crowds. While previous social psychology studies have explored how both males and females formulate long-term group impressions (e.g., Hoxter and Lester, 1994; Ekehammar et al., 2003), our study examined whether there are actual *perceptual* differences between males and females when they evaluate a crowd of faces at first glance. Our data show that compared to males, females have a perceptual advantage in determining average crowd characteristics. It is important to note that the main effect of gender was associated with a small effect size. Nevertheless, since we have replicated the main effect across all the three studies, it is nonetheless a robust finding.

This perceptual advantage in crowd discrimination is likely a result of females' relatively superior individual face identification abilities, which has been discovered by previous research (Rehnman and Herlitz, 2006; McBain et al., 2009; Heisz et al., 2013; Sommer et al., 2013). In a series of experiments, McBain et al. (2009) manipulated both the orientation of the faces (upright vs. inverted) and the information available in the faces (low vs. high noise). They found that females consistently performed better in upright, high-noise face processing tasks by relying on holistic processing skills. Our results extend these findings. Ensemble coding by definition requires participants to gather information about individual faces first, and our study suggests that females' single face advantages may contribute to their ensemble coding performance. Furthermore, females' superior episodic memory and working memory (Herlitz et al., 1997, 1999; Harness et al., 2008) may also contribute to females' better crowd perception. While the relationship between memory and ensemble coding has not been fully explored, previous authors have suggested that perception inherently involves memory (e.g., Luck and Vogel, 1997). Brady and Alvarez demonstrated that ensemble coding influences working memory. In a working memory task, participants reported the size and color of individual items in a display, and they found participants' reports were biased toward the mean of the set of items (Brady and Alvarez, 2011). It may well be that the relationship is reciprocal. Therefore, it is possible that females' superior memory enhances the accuracy of their ensemble percept^4^.

While females demonstrably performed better in perceiving a single face and crowds of faces, our data consistently showed that males also exhibited unique strengths: although males' crowd perception was relatively poor compared to female, males' crowd perception was better than would be predicted from their single face perception. Compared to females, a greater proportion of males performed better in the crowd than in the single face perception condition. How is this paradoxical result possible? Considering that crowds are perceived as a summary statistic or ensemble (Haberman and Whitney, 2007), the pattern may not be surprising. For instance, prosopagnosics, individuals who have difficulty discriminating individual faces, still achieved an accurate ensemble percept (Leib et al., 2012). Prosopagnosics' precision in ensemble coding may be a result of noise reduction. In any mathematical averaging process, noise is reduced with a greater number of samples, and greater precision is achieved (Alvarez, 2011; Robitaille and Harris, 2011). Our male subjects' individual face representations were noisy (at least compared to females); however, averaging larger sample sizes (i.e., crowds of faces) may increase precision via noise cancellation. Thus, by integrating more faces into the ensemble precept, males may compensate for relatively poor sensitivity to single faces and improve their perception of the crowd average.

From a typical social psychological perspective, our findings are reasonable. Darwinian consensus is that the acquisition of any mental power or capacity is the result of evolution. Evolutionary pressure may have contributed to the identity sensitivity for females and crowd familiarity for males. For example, in most human societies, males have historically participated in larger social networks compared to females (for review, see Belle, 1989; Benenson, 1990). In contrast, females have been reported to have more intimate confidants and relationships than males (e.g., Williams, 1959; Powers and Bultena, 1976). It is possible that historically differing social networks may impact males' and females' ensemble coding habits, allowing females to precisely scrutinize single faces and allowing males to integrate a greater number of faces, despite their relatively poor single face and group perception as a whole.

This study is a first step toward examining how social and cognitive factors shape people's group perception, and we were primarily interested in the comparison between male and female participants. However, another potentially interesting avenue of research is exploring the own-gender effect (Herlitz and Lovén, 2013). In our particular experiment, we did not observe the own-gender effect. However, this may be because we tested Asian participants with Caucasian stimuli. Future studies should also investigate how participant ethnicity interacts with stimulus ethnicity.

In the current paper, we emphasize that ensemble coding is not solely a cognitive process, but is also influenced by high-level social factors, such as gender constructs. It will be important for future work to test if culture, a well-discussed social factor, shapes participants' immediate group perception. Previous research has documented culture differences in cognitive processes (Nisbett et al., 2001; Norenzayan et al., 2007; Masuda et al., 2008, 2012; Miyamoto, 2013), in which researchers found that while Asians focus on relationships between a focal object and its context, Westerners focus on an object independently from its context (Masuda and Nisbett, 2001; Kitayama et al., 2003). This perceptual habit difference was also found to lead to face perception differences across the two cultures (Miyamoto et al., 2011). For instance, Masuda et al. (2008) asked participants to distinguish the emotion of faces, and found that Caucasians mainly focus on the target face itself, while Japanese participants took contextual information into account. These findings raise interesting questions for future ensemble coding research. Will Asians be more influenced by the context of the crowd compared to Caucasians? Will Asians, while judging a subset of the crowd, be more influenced by faces outside the subset? Examining the interaction between culture and ensemble coding may yield interesting insights into differences between Westerners' and Easterners' perceptions of crowds.

Across all three studies, we tested group perception using morphed faces because these faces have been well controlled for subtle variations and thus allow us to concretely measure error in participants' average face judgment (Haberman and Whitney, 2007, 2009; Haberman et al., 2009). However, the complex nature of group perception does place limits on the extent to which real life group perception can be examined experimentally and prospectively in a laboratory setting. Using current research as a basis, future research needs to explore using different stimuli (e.g., crowds of emotional faces, crowds with full-body pictures, etc.) and different groups (e.g., people with different ethnicities, people with different social status, etc.). Furthermore, while the current study limits its focus to examining the differences in ensemble coding performance between males and females, it is intriguing to consider whether perceptual differences in crowd evaluation might affect males' and females' impression formation over time and across various social contexts. A number of social psychology studies report that crowd observations, as well as the subsequent schemas that develop, guide future social interactions (e.g., Devine, 1989; Spencer et al., 1999). We suggest that the relationship between perceptual analysis of the crowd and social interaction may be reciprocal. Variations in visual processing influence social evaluations of groups, and, conversely, diversities in social background influence perception. Considering the possible interactions between perceptual and social processes within crowds and their members, an interesting avenue of future investigation will be to evaluate whether an individual's ensemble coding performance influences or is related to social phenomena such as stereotyping, impression formation, category accentuation, prejudice, and intergroup conflicts and cooperation. This future work will illuminate how our perception of the world mediates our social interactions, and how our social identities mediate the processes of social perception.

### Conflict of interest statement

The authors declare that the research was conducted in the absence of any commercial or financial relationships that could be construed as a potential conflict of interest.

## Footnotes

^1^We used a permutation test commonly used in statistics (Good, 2000; Chau et al., 2004). Specifically, we created two permutated groups by randomly shuffling the male and female labels and rearranging paired single face AEE and multi-face AEE. For each permutation, we calculated the slope of each permutated subgroup (permutated “male” and “female”) and subtracted the permutated “male” group's slope from the permutated “female” group's slope. This procedure was repeated 1000 times, yielding a null distribution of slope differences. By comparing the original difference between real male and female slopes against the null distribution, we can determine whether the difference between male and female slopes is significant by calculating the proportion of sampled permutations whose slope difference was greater or equal to the observed gender slope difference.

^2^The randomly chosen faces were still drawn from within a designated range from the mean of the trial.

^3^This is not a realistic estimate but is a conservative estimate. In actuality, participants are probably much noisier (not centered on the perfect average). If this is the case, the results of the simulation would yield a much higher error, suggesting that participants integrated even more faces.

^4^In our particular task, working memory may also play a significant role during the test phase. During this phase, participants must keep in mind the average identity while scrolling through the test faces (147 in all).

## Supplementary material

Supplementary Figure 1 **Display examples for Experiments 2a and 2b. (A)** Each display contained either a single face or 4 identities, each repeating 1, 2, or 3 times. The four faces in the 4, 8, and 12 face conditions were 3 and 9 steps away from the mean face in either direction, with a consistent variance. **(B)** Each display contained 1, 2, 4, or 8 faces randomly selected from 12 faces. Thus, the variance of the displayed faces was not restricted. Note: all the faces were randomly chosen and randomly displayed on the screen.

Supplementary Video 1 **Two trials for Experiment 1**. Participants were presented with 18 faces on the screen before one test face was shown on the screen. By scrolling the mouse, participants could search through the whole face wheel to find the estimated average face.
