# Supplementary material for: Gender differences in crowd perception
Source: Front Psychol. 2015 Sep 2;6:1300. doi: 10.3389/fpsyg.2015.01300 (PMC4557101; doi:10.3389/fpsyg.2015.01300)
Supplement: Supplementary Figure 1 — Display examples for Experiments 2a and 2b. (A) Each display contained either a single face or 4 identities, each repeating 1, 2, or 3 times. The four faces in the 4, 8, and 12 face conditions were 3 and 9 steps away from the mean face in either direction, with a consistent variance. (B) Each display contained 1, 2, 4, or 8 faces randomly selected from 12 faces. Thus, the variance of the displayed faces was not restricted. Note: all the faces were randomly chosen and randomly displayed on the screen. [file Image1.PDF]

Supplementary Figure

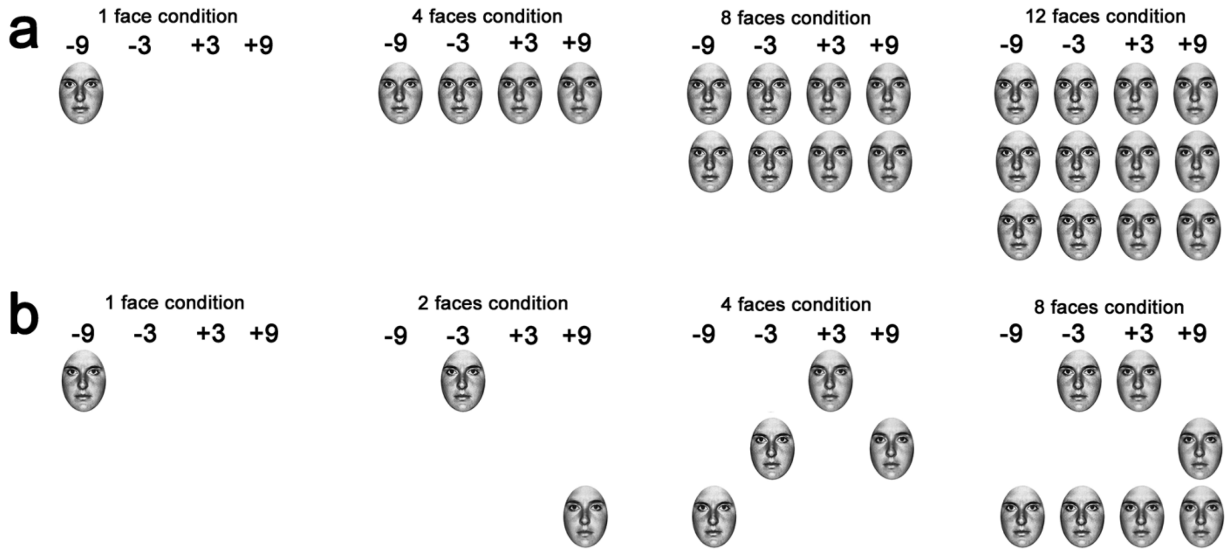

Supplementary Figure. Display examples for Experiments 2a & 2b. (a) Each display contained either a single face or 4 identities, each repeating 1, 2, or 3 times. The four faces in the 4, 8, and 12 face conditions were 3 and 9 steps away from the mean face in either direction, with a consistent variance. (b) Each display contained 1, 2, 4, or 8 faces randomly selected from 12 faces. Thus, the variance of the displayed faces was not restricted. Note: all the faces were randomly picked and randomly displayed on the screen.
