# Supplementary material for: Gender differences in crowd perception
Source: Front Psychol. 2015 Sep 2;6:1300. doi: 10.3389/fpsyg.2015.01300 (PMC4557101; doi:10.3389/fpsyg.2015.01300)
Supplement: Supplementary file 3 [file Image2.PDF]

Supplementary material for Study1

In study 1, our stimuli consisted of 2 arrays of faces. Each array contained 147 faces, generated by morphing together photographs of three different individuals (A, B, and C), each with a neutral expression (Figure 1). One array was morphed from 3 grayscale female photos from the Ekman gallery (Ekman & Friesen, 1976), while the other array was morphed from 3 grayscale male photos from the same gallery. We used the program Morph 2.5 (Gryphon Software, San Diego, CA) to linearly interpolate each of the three pairs of original faces (A and B, B and C, C and A) to create 48 morphs per pair, yielding a "circle" of 147 neutral faces with no end or beginning. The result of this morphing procedure is that the stimuli share many facial features in common, yet are measurably and perceptually distinct from each other. These shared similarities are comparable to family resemblance, or to populations in regions with relatively little genetic variation, with the caveat that laboratory stimuli are systematically controlled. Thus, feature variations in the laboratory stimuli are necessarily subtler compared to faces observed in naturalistic settings; this is a strength rather than a weakness because the faces are more quantifiable and controlled. During the experiment, participants viewed displays of 18 morphed faces chosen from one of the stimulus arrays (each stimulus array contained 147 faces in total). The faces were shown in a grid pattern on the computer screen. In the first 2 blocks, we displayed the faces in an upright orientation, while in the other 2 blocks we displayed the faces in an inverted orientation. Inverted face trials allowed us to determine whether participants were ensemble coding using a feature-based strategy (associated with low-level processing) or a holistic analysis strategy (associated with higher-level cognitive and social processing;Farah, Tanaka & Drain, 1995; Tanaka & Farah, 1993; for review see Maurer, Grand, & Mondloch, 2002). In other words, using inverted stimuli allowed us to determine whether participants were using strategies associated with face processing in everyday life, or whether they were relying on the color or basic features (orientation, texture, etc.) contained within the face to complete the ensemble coding task (a strategy that could theoretically allow the participants to successfully complete the task, but would not be associated with face processing strategies used in daily life (Farah et al., 1995; Haxby et al., 1999;Tanaka & Farah, 1993; for review see Maurer et al., 2002). Each stimulus array also served as a continuum from which participants selected their responses (see further descriptions below in the Procedures section). Each face from the set was 3 cm by 3.7 cm, subtending 2.86×3.53 degrees of visual angle. All stimuli were viewed on a 19 inch monitor with resolution of 1280×1024 pixels with a 75 Hz refresh rate. Subjects performed the experiment in a darkened room, seated at a distance of 80 cm from the monitor.
